# Anandamide Induces Sperm Release from Oviductal Epithelia through Nitric Oxide Pathway in Bovines

**DOI:** 10.1371/journal.pone.0030671

**Published:** 2012-02-17

**Authors:** Claudia Osycka-Salut, María Gracia Gervasi, Elba Pereyra, Maximiliano Cella, María Laura Ribeiro, Ana María Franchi, Silvina Perez-Martinez

**Affiliations:** Centro de Estudios Farmacológicos y Botánicos (CEFYBO-CONICET), Buenos Aires, Argentina; State Key Laboratory of Reproductive Biology, Institute of Zoology, Chinese Academy of Sciences, China

## Abstract

Mammalian spermatozoa are not able to fertilize an egg immediately upon ejaculation. They acquire this ability during their transit through the female genital tract in a process known as capacitation. The mammalian oviduct acts as a functional sperm reservoir providing a suitable environment that allows the maintenance of sperm fertilization competence until ovulation occurs. After ovulation, spermatozoa are gradually released from the oviductal reservoir in the caudal isthmus and ascend to the site of fertilization. Capacitating-related changes in sperm plasma membrane seem to be responsible for sperm release from oviductal epithelium. Anandamide is a lipid mediator that participates in the regulation of several female and male reproductive functions. Previously we have demonstrated that anandamide was capable to release spermatozoa from oviductal epithelia by induction of sperm capacitation in bovines. In the present work we studied whether anandamide might exert its effect by activating the nitric oxide (NO) pathway since this molecule has been described as a capacitating agent in spermatozoa from different species. First, we demonstrated that 1 µM NOC-18, a NO donor, and 10 mM L-Arginine, NO synthase substrate, induced the release of spermatozoa from the oviductal epithelia. Then, we observed that the anandamide effect on sperm oviduct interaction was reversed by the addition of 1 µM L-NAME, a NO synthase inhibitor, or 30 µg/ml Hemoglobin, a NO scavenger. We also demonstrated that the induction of bull sperm capacitation by nanomolar concentrations of R(+)-methanandamide or anandamide was inhibited by adding L-NAME or Hemoglobin. To study whether anandamide is able to produce NO, we measured this compound in both sperm and oviductal cells. We observed that anandamide increased the levels of NO in spermatozoa, but not in oviductal cells. These findings suggest that anandamide regulates the sperm release from oviductal epithelia probably by activating the NO pathway during sperm capacitation.

## Introduction

The mammalian oviduct acts as a functional sperm reservoir providing an environment that allows maintenance and competition for fertilization of the oocyte. In different species, spermatozoa are sequestered in the lower region of the oviduct (isthmus) where they attach to epithelial cells. This event extends the sperm life, delaying sperm capacitation until ovulation-associated signals induce their release allowing the transit to the upper region of the oviduct (ampulla). Adherence to the oviduct plays a key role in the selection of spermatozoa. The binding and release of spermatozoa from the oviductal epithelium are modulated mainly by the sperm capacitation and only non-capacitated spermatozoa bind to oviductal cells [1]–[2].

Sperm capacitation includes a large number of structural and metabolic modifications such as an increase in intracellular ions and protein tyrosine phosphorylation, generation of reactive oxygen species and changes in metabolism, plasma membrane fluidity and motility [3]–[4].

Molecules from oviductal fluid, such as sulphated glycoconjugates like heparin, are involved in regulating sperm-oviduct interactions and bovine sperm capacitation [5]–[8]. The concentrations of those compounds in oviduct luminal fluid are under cyclic ovarian control, reaching a peak during the period of estrus [8].

Some evidences indicate that a number of lipid mediators serve as important signaling molecules during fertilization and early pregnancy. Among these lipid messengers, prostaglandins, eicosanoids generated from arachidonic acid by cyclooxygenases and lysophosphatidic acid that belongs to the lysophospholipid group, are well recognized signals in reproductive events [9]–[10]. So far there is little information about the participation of lipid molecules in sperm-oviduct interactions.

The N-arachidonoylethanolamide or anandamide (AEA) is an endogenous lipid agonist of cannabinoid receptors (CB1 and CB2; [11]–[12]) or vanilloid receptor type 1 (TRPV1; [13]). AEA is released from membrane phospholipids of neurons and other cells stimulated by depolarizing agents. Once released, AEA effect is quickly terminated by membrane-bound fatty acid amide hydrolase (FAAH; [14]), suggesting a critical role for this lipid during cell signaling. The endocannabinoid system has recently been characterized in both oviduct and sperm cells of mammals [15]–[18]. In addition, significant levels of AEA are found in seminal plasma, mid-cycle oviductal fluid, and follicular fluid [19]–[21]. Previously we demonstrated that bovine oviduct and bull spermatozoa express the endocannabinoid system and that nanomolar concentrations of AEA or its non-hydrolysable analog, R(+)-methanandamide (MetAEA), regulate sperm release from the oviductal epithelial cells [22]. Furthermore we have recently found that AEA is capable to induce sperm capacitation by activation of CB1 and TRPV1 receptors but not by CB2 and that it could be involved in the same molecular pathway as heparin in bovines [23].

Signal-transduction pathways involving AEA include modulation of the adenylate cyclase, the activation of mitogen-activated protein kinase and cytosolic phospholipase A_2_, activation/inhibition of ionic currents, modulation of intracellular Ca^2+^ concentration and regulation of nitric oxide (NO) synthases [24]–[25].

Nitric oxide is a short-life free radical synthesized by NO synthases (NOS) that are responsible for the conversion of L-Arginine to L-citrulline and NO [26]. Three isoforms have been detected in different female reproductive tissues including the bovine oviduct [27]–[29]. The endothelial and neuronal NOS isoforms have been observed in mouse [30]–[31], human [30] and bovine spermatozoa [32].

Nitric oxide appears to be involved in sperm and oviductal functions [4], [33]–[34]. Previous reports revealed that mammalian spermatozoa exhibit NOS activity (boar: [35]; human: [36]; bull: [32]) and that NO acts as an intracellular signaling molecule in sperm capacitation and acrosome reaction [4], [35], [37].

Several works show the interaction between AEA and NO pathways [38]–[40]. Low levels of AEA produced a sustained release of NO in the endothelium through the induction of TRPV1 [41]. The interaction between NO and AEA pathways was also observed in reproductive functions. In rat placenta, AEA modulates NO levels by two independent ways: 1) decreasing NOS activity by activation of CB receptors and 2) stimulating NO synthesis by TRPV1 activation [42].

Previous evidences from us and other groups indicate that 1) AEA signaling regulates sperm-oviduct interaction in bovines [22] and induces bull sperm capacitation [23], 2) cannabinoid receptors activate signaling pathways that control intracellular NO levels [43] and 3) NO promotes bovine sperm capacitation [37]. The activation of cannabinoid receptors may increase NO levels stimulating sperm capacitation and/or sperm release from oviductal reservoir. Therefore, the aim of this work was to study the possible interaction between the endocannabinoid and nitrergic systems in the regulation of the sperm release from the oviduct and sperm capacitation in bovines. To demonstrate this, we studied 1) whether NO, by AEA induction, induces sperm release from oviductal epithelial cells, 2) whether AEA is able to induce sperm capacitation by activating the NO pathway, 3) whether AEA is able to modulate NO levels in both spermatozoa and oviductal epithelia.

## Results

### Effect of a NO donor and L-Arginine in sperm-bovine oviductal epithelial cells (BOEC) interaction

**Figure 1:**
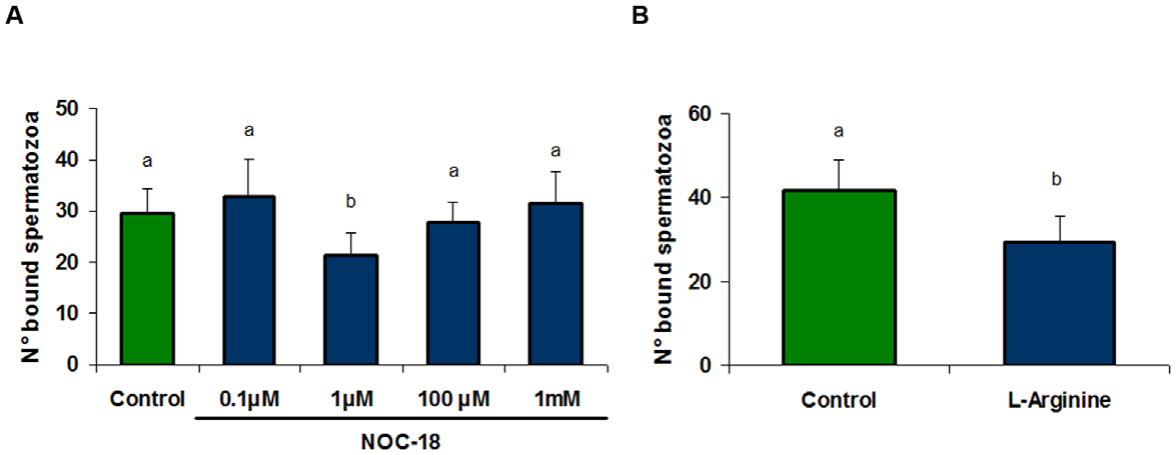
Assessment of the participation of nitrergic system on sperm release from bovine oviductal monolayers (BOEC). Sperm cells and BOEC were co-cultured and then incubated for 15 min with BSA-free sp-TALP alone (control), increasing concentrations of A) NOC-18 (a NO donor) or B) L-Arginine (10 mM; NO synthase substrate). Bars indicate the number of spermatozoa that remained attached to the monolayers and represent the mean ± S.E.M. of bound spermatozoa/0.11 mm^2^ monolayer (n = 6), a≠b p<0.05.

To study the participation of the NO in the release of the spermatozoa from oviductal epithelia, co-cultures of BOEC and sperm cells were incubated with increasing concentrations of NOC-18, a NO donor. Results are presented in Figure 1A and indicate that NOC-18 produced 30% of sperm release from BOEC compared to control. Low or high concentrations did not exert any effect.

To corroborate this result we analyzed whether L-Arginine, the NOS substrate, might produce a similar effect. The incubation with L-Arginine reduced the number of bound-sperm to oviductal cells respect to control (Figure 1B).

### Interaction of AEA and NO in the regulation of sperm release from BOEC

We have previously shown that AEA and MetAEA induced sperm release from oviductal epithelia in bovines [22].

**Figure 2:**
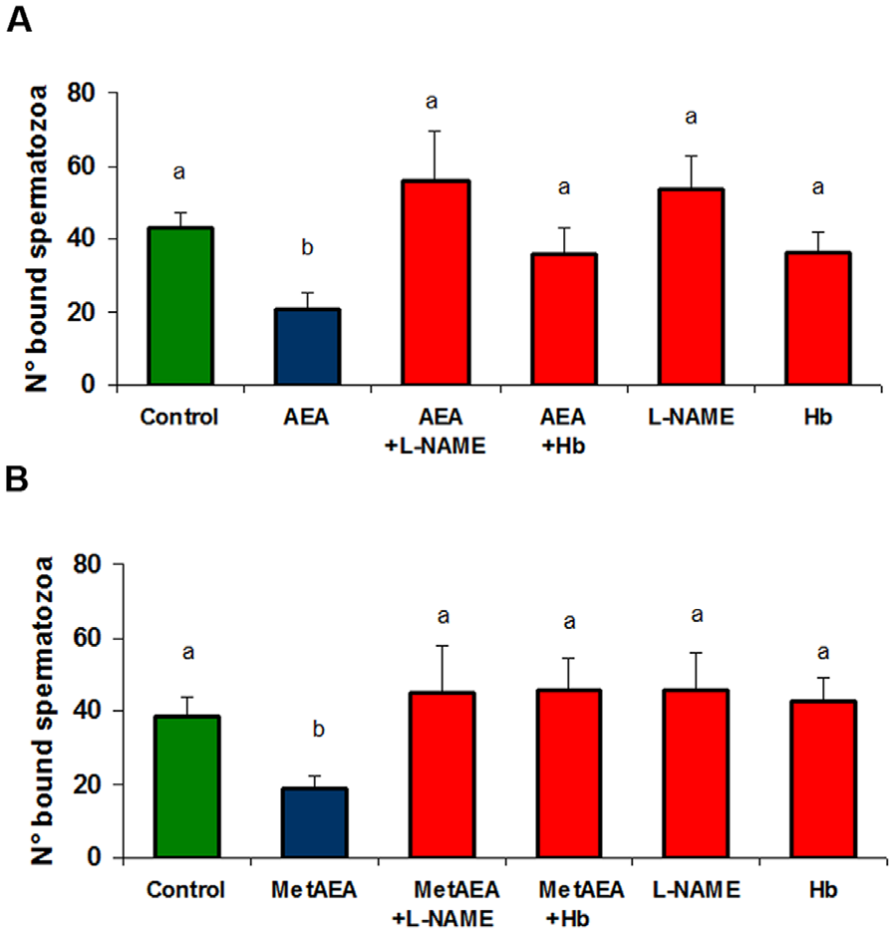
Assessment of participation of the nitrergic system on sperm release induced by anandamide. Sperm cells and BOEC were co-cultured and then incubated for 15 min with BSA-free sp-TALP alone (control), AEA (1 nM) or MetAEA (1.4 nM) and L-NAME (1 µM; NO synthase inhibitor) or Hemoglobin (Hb) (30 µg/ml; NO scavenger). Bars indicate the number of spermatozoa that remained attached to the monolayers and represent the mean ± S.E.M. of bound spermatozoa/0.11 mm^2^ monolayer (n = 6), a≠b p<0.05.

Since our results suggested that NO might regulate the sperm-BOEC interaction, we investigated whether nitrergic and endocannabinoid systems could be interacting in the regulation of this process. Thus, we performed sperm release experiments co-incubating AEA or MetAEA with L-NAME (a non-selective NOS inhibitor). As previously demonstrated [22], AEA and MetAEA produced 40% decrease in the number of bound spermatozoa respect to control samples. Interestingly, L-NAME reversed the effect of AEA (Figure 2A) and MetAEA (Figure 2B), and the number of bound spermatozoa to BOEC was equal to that observed in controls. In addition, the incubation with Hemoglobin (Hb, a NO scavenger) also inhibited the effect of both cannabinoid agonists (Figure 2A and 2B). L-NAME and Hb alone did not exert any effect on sperm-BOEC interaction.

### Interaction of AEA and NO on sperm capacitation

As mentioned in the introduction, after ovulation, spermatozoa are gradually released from the reservoir in the caudal isthmus and ascend to the ampulla. Changes related to capacitation in sperm plasma membrane seem to be responsible for sperm release from oviduct [44].

Previous results from our own indicated that AEA and MetAEA induce bull sperm capacitation [23]. In this work we investigated whether AEA could exert capacitating effects through the signaling pathway that involves NO. The sperm capacitation related-events were evaluated by CTC assay and LPC-induced acrosome reaction (AR) assessed by PSA-FITC.

**Figure 3:**
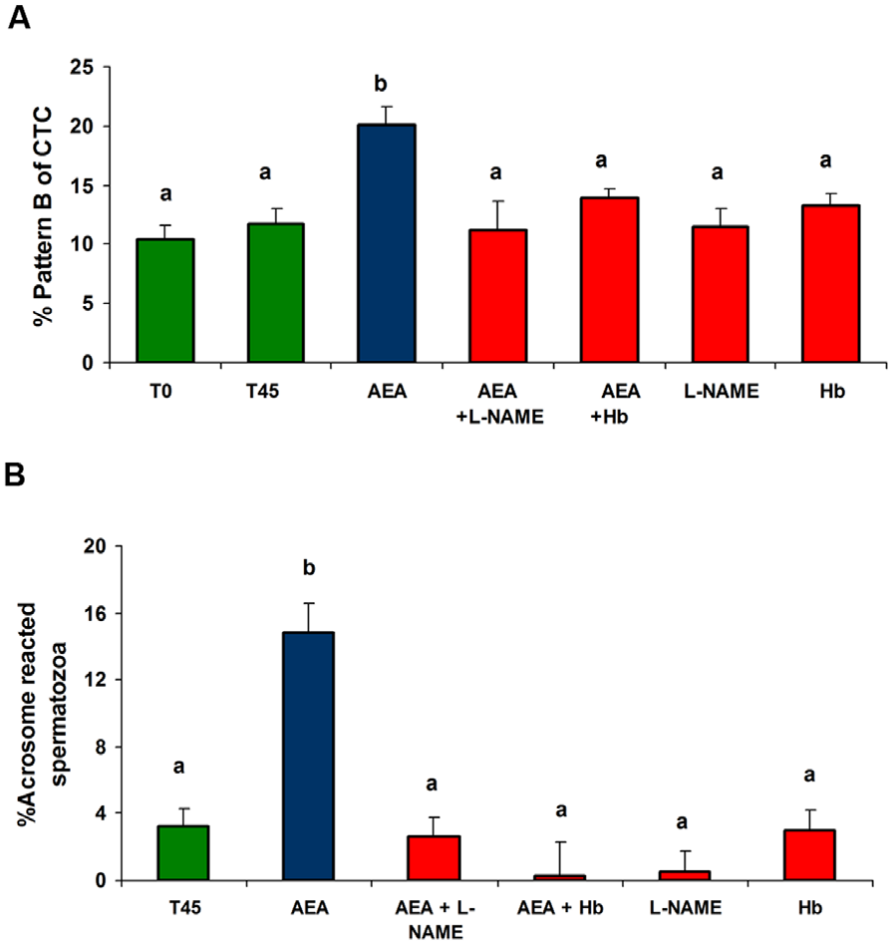
Effect of L-NAME or Hemoglobin on bull sperm capacitation induced by AEA. Spermatozoa were incubated for 45 min at 38.5°C in 0.3% BSA sp-TALP medium with AEA (1 nM) and L-NAME (1 µM) or Hb (30 µg/ml). Bars indicate the percentage of capacitated spermatozoa (A: % pattern B of CTC; B: % acrosome reacted spermatozoa). **A:** Assessment of sperm capacitation by CTC; T0 and T45: 0.3% BSA sp-TALP at 0 and 45 min (control) incubation respectively (n = 6). **B:** Assessment of sperm capacitation by LPC-induced acrosome reaction (AR)-PSA-FITC. T45: 0.3% BSA sp-TALP (control). After capacitation, spermatozoa were incubated for 15 min either with or without LPC to induce AR. Bars show percentage of spermatozoa that underwent LPC-induced AR minus the percentage of spermatozoa that underwent spontaneous AR (n = 6). Data are expressed as mean ± SEM. a≠b, p<0.05.

**Figure 4:**
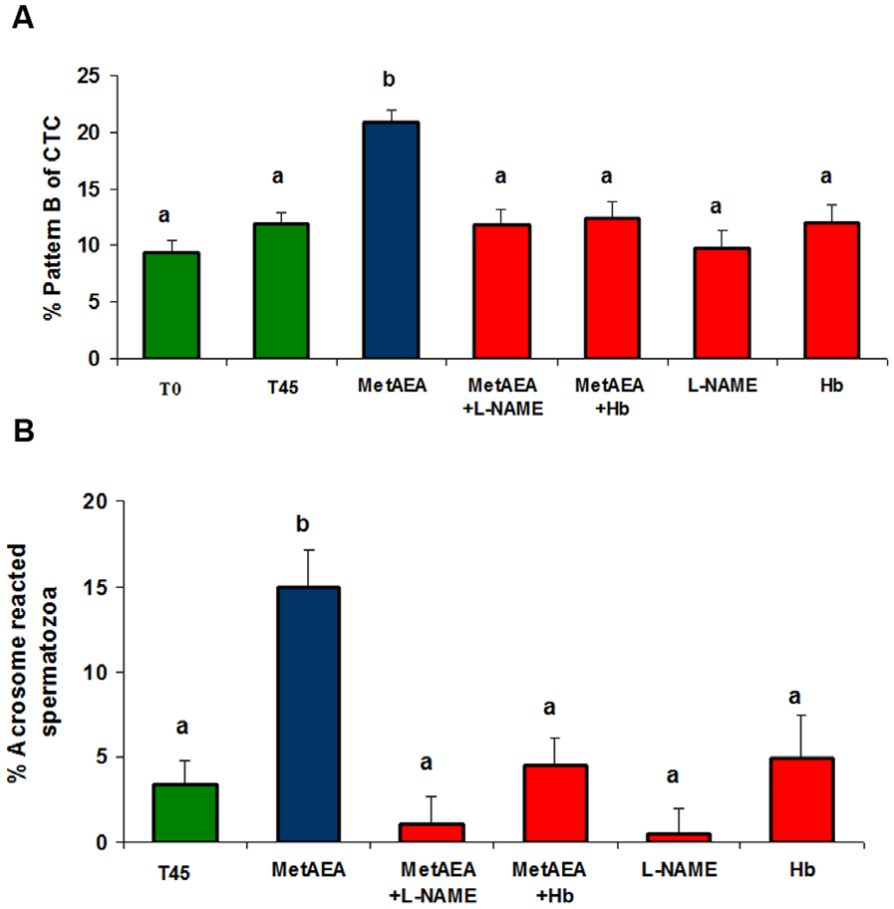
Effect of L-NAME or Hemoglobin on bull sperm capacitation induced by MetAEA. Spermatozoa were incubated for 45 min at 38.5°C in 0.3% BSA sp-TALP medium with MetAEA (1.4 nM) and L-NAME (1 µM) or Hb (30 µg/ml). Bars indicate the percentage of capacitated spermatozoa (A: % pattern B of CTC; B: % acrosome reacted spermatozoa). **A:** Assessment of sperm capacitation by CTC; T0 and T45: 0.3% BSA sp-TALP at 0 and 45 min (control) incubation respectively (n = 6). **B:** Assessment of sperm capacitation by LPC-induced acrosome reaction (AR)-PSA-FITC. T45: 0.3% BSA sp-TALP (control). After capacitation, spermatozoa were incubated for 15 min either with or without LPC to induce AR. Bars show percentage of spermatozoa that underwent LPC-induced AR minus the percentage of spermatozoa that underwent spontaneous AR (n = 6). Data are expressed as mean ± SEM. a≠b, p<0.05.

As shown in Figure 3 (A and B) and Figure 4 (A and B), consistent with the sperm releasing results, the capacitating effect of cannabinoid agonists was significantly inhibited by L-NAME.

We also observed that the induction of capacitation by AEA and MetAEA was abolished by the addition of Hb, a NO scavenger (Figures 3A,3B and Figures 4A,4B). The incubation with L-NAME or Hb alone did not have any effect.

Sperm viability and progressive motility of bull spermatozoa were unaffected in the presence of NOC-18, L-Arginine, AEA, MetAEA, L-NAME or Hb (data not shown).

### Effect of MetAEA on NO production

The results so far suggest that both MetAEA and AEA induce sperm capacitation through the activation of the pathway involving NO and could promote the sperm release from oviductal epithelia. Therefore, we studied whether AEA was able to increase NO levels in both sperm and in bovine oviductal cells. As AEA and MetAEA produced similar effects and the last one is a non-hidrolysable AEA analog, the following experiments were performed with MetAEA.

### Assessment of NO levels in bovine spermatozoa

**Figure 5:**
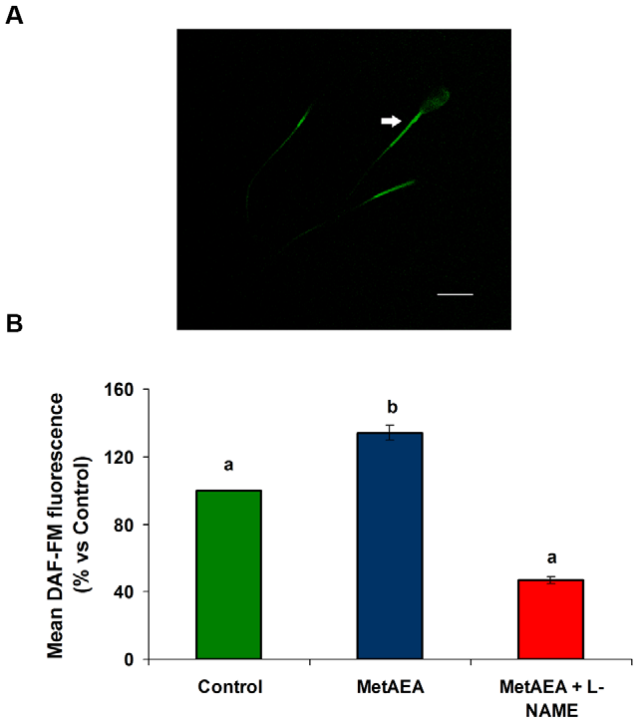
Determination of NO levels in bull spermatozoa. Sperm samples were incubated for 60 min at 38.5°C in 0.3% BSA sp-TALP containing 0.1 mM L-Arginine and 5 µM of DAF-FM diacetate and untreated (control) or treated with MetAEA (1.4 nM), MetAEA+L-NAME (1 µM). Spermatozoa were fixed and the fluorescent complex was measured by flow cytometry. A) A representative photograph showing fluorescence in spermatozoa, indicating the content of intracellular NO (Magnification, ×600); B) Fluorescence data are expressed as mean fluorescence (percentage of control at 45 min incubation, control adjusted to 100%). Data are expressed as mean ± SEM (n = 8). a≠b, p<0.05.

To study whether AEA modulates NO levels during capacitation, spermatozoa pre-loaded with DAF-FM diacetate (Figure 5A) were incubated in sp-TALP with or without MetAEA. After 60 min incubation without capacitating stimulus, we detected fluorescence in spermatozoa indicating that a basal production of NO occurs constitutively in these cells.

A significant increase of NO-DAF-FM fluorescence was observed in spermatozoa capacitated with MetAEA (Figure 5B), which indicates a NO accumulation after 60 min of incubation. MetAEA-stimulated NO-DAF-FM fluorescence was attenuated by L-NAME.

### Participation of CB1 and TRPV1 receptors in the NO production induced by MetAEA

**Figure 6:**
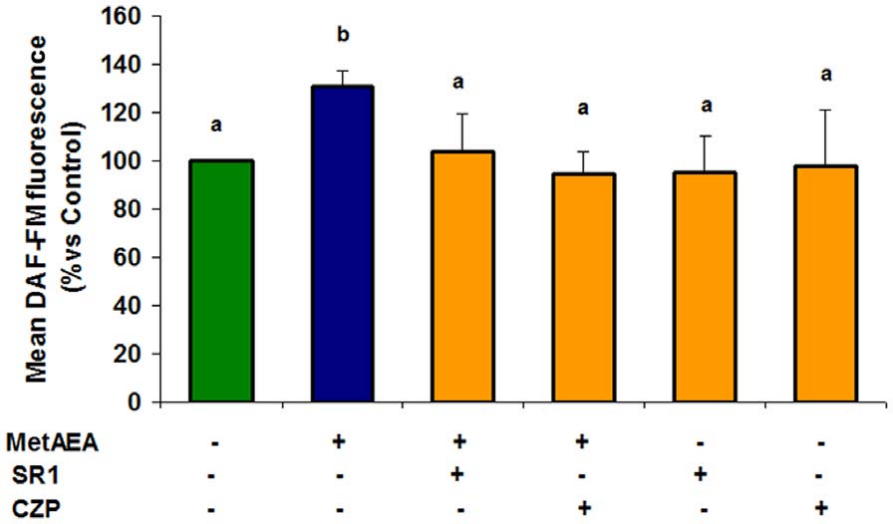
Participation of CB1 and TRPV1 in NO production during bull sperm capacitation by AEA. Sperm samples were incubated for 60 min at 38.5°C in 0.3% BSA sp-TALP containing 0.1 mM L-Arginine and 5 µM of DAF-FM diacetate and untreated (control) or treated with MetAEA (1.4 nM) and/or SR141716A (SR1: CB1 antagonist (0.1 nM)) or Capsazepine (CZP: TRPV1 antagonist (10 nM)). Spermatozoa were fixed and the fluorescent complex was measured by flow cytometry. Fluorescence data are expressed as mean fluorescence (percentage of control at 45 min incubation, control adjusted to 100%). Data are expressed as mean ± SEM (n = 5). a≠b, p<0.05.

In a previous report we demonstrated that AEA induced sperm capacitation by CB1 and TRPV1 activation [23]. Therefore, in this work we investigated whether the increase of sperm NO levels by AEA may occur through those receptors. The results are shown in Figure 6 and indicate that the induction of NO in capacitated spermatozoa by AEA was reversed by both SR141716A and Capsazepine, CB1 and TRPV1 antagonists respectively. The incubation with the antagonists alone had no effect.

### Assessment of NO levels in bovine oviductal cells

The assessment of NO from oviductal cells was performed by two techniques: Griess assay and NOS activity.

The NO_2_^−^/NO_3_^−^production in the culture supernatants was similar when oviductal cells were incubated with or without MetAEA (Table 1a).

**Table 1:** Measurement of NO levels in bovine oviductal cells.

Treatments	(a) NO_2_^−^ concentration(µM NO_2_^−^/µg protein)	(b) NOS activity(fmol citrulline/µg protein ×15 min)
Control	0.52±0.16	2.38±0.90
MetAEA	0.57±0.17	2.43±0.80

**(a) Assessment of NO_2_^−^/NO_3_^−^ production.** Oviductal cultures were incubated for 60 min at 38.5°C and 5% CO_2_ in the presence of M199 medium (control) or MetAEA (1.4 nM). Then, once removed the culture medium, cells were incubated in M199 for 24 h at 38.5°C with 5% CO_2_ to allow the accumulation of NO_2_^−^ and NO_3_^−^ in culture supernatants. NO production (NO_3_^−^ plus NO_2_^−^) was measured using the Griess assay; (n = 26; p>0.05).

**(b) Assessment of NO synthase activity.** Confluent cell monolayers were incubated for 30 min at 38.5°C and 5% CO_2_ in the presence of: 1) M199 medium (control) or 2) MetAEA (1.4 nM). Control and treated cells were removed by trypsinization, centrifuged and incubated for 15 min at 37°C in a HEPES buffer containing L-[^14^C]-Arginine and NADPH (see material and methods); (n = 7; p>0.05).

Data are expressed as mean ± SEM.

NOS activity was performed in oviductal epithelia cells incubated with MetAEA. The incubation with MetAEA did not increase NOS activity in oviductal cells (Table 1b), these results are in accordance with NO_2_^−^/NO_3_^−^ measurements.

### Immunolocalization of NO synthase in bovine cryopreserved spermatozoa

**Figure 7:**
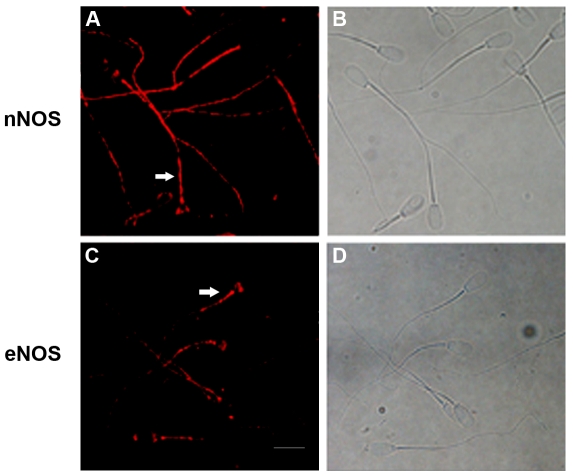
Localization of nNOS and eNOS in bovine spermatozoa. Spermatozoa were incubated with the primary antibodies against nNOS (panel A) or eNOS (panel C); B and D) phase contrast. Controls were performed incubating sperm cells with IgG fractions from non-immunized rabbits at the same concentration that the primary antibody (data not shown) (n = 3); Scale bar: 10 µm (Magnification, ×600). Arrows indicate the immunoreactive staining of the NOS antibodies.

Previously, Meiser and Schulz [32] demonstrated the expression of the two constitutive NOS isoforms in spermatozoa from ejaculated bovine semen. To investigate which isoforms are present in cryopreserved bovine sperm, immunocytochemical experiments were performed. The results are shown in Figure 7 and indicate that cryopreserved bull sperm expressed nNOS and eNOS isoforms. The analysis of the images indicated that both isoenzymes were mainly located in the tail of the spermatozoa (mid and main piece) and some spermatozoa were stained in the post-acrosomal region. Incubation with two different antibodies against iNOS isoform showed a non defined localization so we could not confirm the presence of this isoform in bovine sperm (data not shown).

## Discussion

In the present work the participation of NO in the regulation of sperm-oviduct interaction in bovines is demonstrated. The incubation with NOC-18 (a NO donor) or L-Arginine (the NOS substrate) of sperm-BOEC co-cultures induces sperm release from oviductal cells.

Interestingly, only 1 µM NOC-18 is effective and lower or higher concentrations have no effect. At high concentrations, NO or its derivatives such as ONOO^−^, may have a deleterious effects on cellular functions by acting on membrane components such as lipids or molecules with thiol groups, as previously described by Rodriguez et al. [45]. At low concentrations, NO levels might be insufficient to induce the release.

The induction of sperm capacitation by L-Arginine at a concentration of 10 mM without affecting the viability and motility in cryopreserved bovine spermatozoa is well documented [46]. Therefore, L-Arginine favors sperm capacitation, and thus could facilitate through this process the sperm release from the oviductal epithelial cells. Those results also suggest that NO *per se*, may modulate and participate in the release of spermatozoa from their oviductal reservoir.

Taking into account previous results of our laboratory [22], we investigated whether AEA-mediated release involves the NO pathway. Here, we find that NO is an intermediate of this pathway, since the effect of AEA and its stable analog, MetAEA, is reversed by a NOS inhibitor or by a NO scavenger.

Spermatozoa exposed to capacitating conditions have a limited ability to attach to oviductal cells *in vitro* [47]. In addition, non-capacitated spermatozoa initially bind to oviductal epithelial cells both *in vivo* [48] and *in vitro* [49]. This initial attachment is followed by induction of capacitation and release of capacitated sperm [48]–[49]. From these evidences and taking into account that AEA is a capacitating agent in bovine [23] as well as NO [37], we investigated whether AEA could induce sperm capacitation by activating the NO pathway.

The capacitating effect of AEA and MetAEA is inhibited by L-NAME or Hb, suggesting that NO might interact with the AEA pathway in this process. Previous evidences, recently described in our laboratory, indicate that AEA is involved in sperm capacitation induced by heparin, a glycosaminoglycan known as bull sperm capacitation inducer [23]. Besides, Rodriguez et al. [37] found that heparin-induced capacitation is inhibited by the presence of a NOS inhibitor. Further experiments will allow us to establish whether a possible shared-pathway between heparin and AEA in the activation of the NO pathway occurs during sperm capacitation in cattle.

In agreement with our results so far presented, other authors demonstrated that heparin is a fast and highly effective inductor of sperm release from oviductal epithelial cells *in vitro* [5], [50]. This might reflect changes or loss of binding molecules on the sperm plasma membrane promoting sperm release.

Though potential sources of NO are present throughout the female reproductive tract, it is probable that, in our system, AEA activates sperm NO/NOS pathway as part of the molecular mechanisms activated in sperm capacitation.

Bovine oviduct expresses the three NOS isoforms and the relevant role of NO in the regulation of the oviductal function is widely demonstrated [33], [51]. In addition, such molecules as ovarian hormones regulate NO synthesis in the mammalian oviduct. In this work, AEA does not modulate NO production in bovine oviductal cells. However, during sperm capacitation after DAF-FM loading, MetAEA induces an increase in fluorescence in the neck/midpiece of most cells, indicative of NO production. The site of NO synthesis is consistent with the localization of nNOS and eNOS isoforms found in the bovine cryopreserved spermatozoon (see Figure 7). The addition of L-NAME decreases NO levels suggesting that AEA was acting specifically on NOS.

We have previously found that bovine sperm capacitation is stimulated by AEA through CB1 and TRPV1 activation because AEA effects were fully antagonized by SR141716A (a CB1 antagonist) or by capsazepine (a TRPV1 antagonist), respectively. Our results indicate also that CB2 is not involved in the induction of bull sperm capacitation elicited by AEA because CB2 antagonist did not reverse the effect of the endocannabinoid [23]. In this work we demonstrated that AEA increased NO sperm levels through CB1 and TRPV1, the receptors that are involved in sperm capacitation induced by this endocannabinoid. The increase of NO production by AEA has been reported by several groups. Ghasemi et al. [52] observed in diabetic rats that AEA could affect erectile function by increasing levels of NO, via CB1 and TRPV1. In turn, AEA acts as a proinflammatory molecule in deleterious effects produced by lipopolysaccharide in implantation sites, increasing NO synthesis via activation of CB1 receptor [40].

As mentioned in the introduction, there is evidence indicating that AEA is present in human mid-cycle oviductal fluid [19]. Also plasma concentration of AEA positively correlated with levels of estradiol and gonadotropins during different stages of the menstrual cycle, suggesting that they would be involved in the regulation of AEA levels in women [20]. These results and the evidence showed in this paper let us propose a model for regulating the sperm-oviduct interaction by the AEA-NO pathway. At the peri-ovulatory period, stimuli such as ovarian hormones could induce an increase in the concentration of AEA in the oviductal tract. Endocannabinoids activate sperm cannabinoid receptors increasing endogenous production of NO promoting sperm capacitation and thus the release of stored spermatozoa in the oviductal reservoir. Another possibility is that ovarian hormones surge during the peri-ovulatory period may also induce the AEA synthesis inside the spermatozoa thus activating the NO pathway in gametes.

In summary, we demonstrated the interaction of the endocannabinoid and the nitrergic systems in the regulation of sperm release from oviductal epithelium and sperm capacitation in bovines. Also, we observed for the first time NO production in bovine spermatozoa capacitated with AEA.

## Materials and Methods

### Chemicals

MetAEA and AEA, M199 medium, bovine serum albumin (BSA), Hemoglobin (Hb), L-Arginine, NG-nitro-L-Arginine methyl ester (L-NAME), chlortetracycline (CTC), *Pissum sativum* agglutinin-FITC staining (PSA-FITC), Hoescht 33258 (H258), L-α-lysophosphatidylcholine (LPC), nitrate reductase, lactate dehydrogenase, NADPH and IgG fractions non-immune rabbits (IgG fraction) serum were from Sigma Chemicals (St. Louis, MI, USA). NOC-18 and DAF FM were from Cayman. eNOS, nNOS and iNOS antibodies were purchased from Cayman and Alexa-Fluor555 goat anti-rabbit IgG was from Molecular Probes (Invitrogen, Carlsbad, CA, USA). [^14^C]L-Arginine (specific activity 360 mCi/mmol) was from Amersham Corp. (Arlington Heights, IL, USA). Dowex AG 50W-X8 cation exchange resin was obtained from Bio-Rad Laboratories (Alfatron, SRL, BsAs, Argentina). All other chemicals were of analytical grade.

### Culture media

Oviduct handling and development of monolayer cultures was performed with medium M199 as described previously in Gervasi et al. [22]. Sperm handling and co-culture experiments were performed with Tyrode bicarbonate buffered medium (sp-TALP, [7]) without BSA (BSA-free sp-TALP). The sperm viability was not affected without BSA because the gametes were washed and immediately co-incubated with oviductal cells.

In sperm capacitation experiments, sperm incubations were performed with sp-TALP supplemented with 0.3% BSA [23].

NOC-18 was incubated during 60 min at 37°C in sp-TALP to reach a stable concentration of release.

### Sperm preparation

Frozen bovine semen from six bulls (20–25×10^6^ spermatozoa/0.5 ml straw), obtained from CIALE (Artificial Insemination Center La Elisa, Buenos Aires, Argentina), CIAVT (Artificial Insemination Center Venado Tuerto, Santa Fé, Argentina) and Cabaña Las Lilas (Buenos Aires, Argentina) was used. Straws were thawed in a water bath (37°C for 30 s). Spermatozoa were subjected to sperm selection using glass wool columns [23] and washed by centrifugation at 800 g with BSA-free sp-TALP.

Pellets were assessed for sperm concentration and motility using a hemocytometer mounted on a microscope stage heated at 38°C.

### Evaluation of sperm motility

Progressive motility was evaluated by light microscopy (Magnification, ×300) on a heat platen (38°C) by the same observer, after each treatment. At least 200 spermatozoa were counted in each sample.

### Oviductal cell cultures

Cultures of oviductal epithelia, obtained from oviducts purchased gently by Río de la Plata slaughterhouse, were prepared as described previously by Gervasi et al. [22]. Briefly, oviducts were collected at the time of slaughter, transported at 4°C, cleaned off surrounding tissues and washed thrice in sterile PBS at 4°C. After that, they were cut, flushed with sterile PBS and squeezed by pressure with tweezers. Laminae of BOEC were recovered from different animals, selected on the basis of ciliary beating, and pooled together. Immunocytokeratin staining was performed to confirm 90% epithelial cell content in the pools. BOEC were washed by centrifugation at 1500 g for 5 min and incubated in M199 medium at 38.5°C in a 5% CO_2_ atmosphere. Incubations were performed in six-well tissue culture dishes with 12 mm round cover slips on the well bottom. After 48 h, BOEC were washed by centrifugation (1500 g for 5 min) and seeded again in tissue dishes. M199 medium was changed every 48 h. Once cell confluence was attained, oviductal monolayers from the same pool of animals were washed thrice in BSA-free sp-TALP and left in this medium for 60 min until sperm addition.

### NO and AEA participation in sperm–oviduct interaction: experimental designs

Co-cultures of BOEC and spermatozoa were performed as described previously by Gervasi et al. [22]. Within each experiment, confluent BOEC monolayers from pooled oviducts were inseminated with the same sperm suspension (0.5–1×10^6^ sperm/ml of BSA-free sp-TALP/well) for 60 min at 38.5°C in a 5% CO_2_ atmosphere. At the end of the co-cultures, unbound sperm populations were removed by washing thrice with BSA-free sp-TALP.

Experiment 1 was performed to evaluate the effect of increasing concentrations NOC-18 (0.1 µM; 1 µM; 100 µM y 1 mM; a NO donor) or 10 mM L-Arginine (NOS substrate) on sperm release from oviductal monolayers.

Experiment 2 was performed to evaluate the effect of L-NAME (1 µM; NOS inhibitor) or Hemoglobin (Hb) (30 µg/ml; NO scavenger) on sperm release induced by AEA (1 nM) or MetAEA (1.4 nM).

Wells containing BOEC monolayers were inseminated with 0.5×10^6^ spermatozoa/ml for 60 min and afterwards unbound sperm were washed. The compounds (NOC-18, L-Arginine, AEA or MetAEA, L-NAME or Hb) were added to the co-cultures for 15 min. Control and treated wells were washed to remove unbound sperm, fixed in glutaraldehyde 2.5% v/v for 60 min at room temperature (RT), extensively washed and mounted on a glass slide. The number of bound sperm was determined by analyzing 20 fields of 0.11 mm^2^/cover slip under a phase contrast microscope (Magnification, ×300).

### *In vitro* spermatozoa capacitation

Ten to 15×10^6^ spermatozoa/ml were placed in 0.3% BSA sp-TALP at 38.5°C and 5% CO_2_ atmosphere for 45 min [23], [46], [53] with MetAEA (1.4 nM), AEA (1 nM), L-NAME (1 µM) and Hb (30 µg/ml). Capacitation was assessed by two techniques: chlortetracycline (CTC) assay and lysophosphatidylcholine (LPC)-induced acrosome reaction assessed by *Pissum sativum* agglutinin-FITC staining.

CTC assay was carried out as described previously by Gervasi et al. [23] and Ward and Storey [54]. The assessment of sperm capacitation was evaluated by the ability of spermatozoa to display CTC fluorescence pattern B, indicative of capacitating status [54]–[55]. Pattern B evaluation was performed on initial sperm suspensions (T0) and after 45 min (T45) of incubation under the conditions mentioned above.

The induction of acrosome reaction was performed as described previously [56] with modifications [23]. Spermatozoa were incubated with the agents for 45 min and then divided in two aliquots of 200 µl; one of them was incubated with LPC (100 µg/ml) and the other without it for 15 min at 38.5°C. To assess viability and acrosome reaction, spermatozoa were incubated with H258 (2 µg/ml) for 5 min, fixed (1% w/v paraformaldehyde) for 8 min at RT and washed with PBS. An aliquot was air-dried onto slides and permeabilized in methanol for 10 min at 4°C. Slides were incubated with 50 µg/ml PSA-FITC for 60 min at RT [23], [57]. At least 200 stained cells/treatment were scored in an epifluorescence microscope. The percentage of capacitated spermatozoa was represented by the difference between percentages of viable-acrosome-reacted spermatozoa in LPC-treated and in non-LPC-treated samples.

### Measurement of NO production in sperm cells

Intracellular NO was monitored with DAF-FM diacetate, a pH-insensitive fluorescent dye that emits increased fluorescence after reaction with an active intermediate of NO formed during the spontaneous oxidation of NO to NO_2_^−^ [58]. Ten to 15×10^6^ spermatozoa/ml were incubated for 60 min at 38.5°C in 0.3% BSA sp-TALP containing 0.1 mM L-Arginine and 5 µM of DAF-FM diacetate (Invitrogen, Carlsbad, CA) and untreated (control) or treated with MetAEA (1.4 nM) or MetAEA+L-NAME (1 µM). In another set of experiments spermatozoa were incubated (in those conditions described below) with MetAEA (1.4 nM), MetAEA+SR141716A (0.1 nM) or MetAEA+Capsazepine (10 nM). After incubation, samples were washed by centrifugation for 10 min at 800 g and the sperm cells were fixed with 4% paraformaldehyde for 60 min at RT. Later, spermatozoa were rinsed two times with PBS, and stored at 4°C until fluorescence was measured by flow cytometry, using a FACSCaliburTM cytometer (BD Pharmigen) to quantify fluorescence (excitation wavelength 488 nm and emission wavelength 530 nm) at the single-cell level, and data were analyzed using Cyflogic 1.2.1 (CyFlo Ltd) software. The autofluorescence control was made incubating with 0.3% BSA sp-TALP without DAF-FM and the control of the basal NO production was made with 0.3% BSA sp-TALP + DAF-FM.

### Assessment of NO production in BOEC cultures

Oviductal cultures were incubated for 60 min at 38.5°C and 5% CO_2_ in the presence of M199 medium (control) and MetAEA 1.4 nM. After that, the culture medium was removed and replaced in all cases by M199 medium. Cells were incubated for 24 h at 38.5°C with 5% CO_2_ to allow the accumulation of nitrates (NO_3_^−^) and nitrites (NO_2_^−^) in culture supernatants.

NO production (NO_3_^−^ plus NO_2_^−^) was measured using the technique described by Grisham et al. [59]. Briefly, culture supernatants, 2 mmol/l NADPH and 10 IU/ml *Aspergillus niger* nitrate reductase were allowed to react in flat-bottomed 96-well culture plates with gentle mixing for 30 min at RT. Then, 100 mmol/l pyruvic acid and 1000 IU/ml lactate dehydrogenase were added and incubated for 10 min. Later, 10 mg/ml sulphanilic acid was added and incubation continued for 10 min. Finally, 1 mg/ml naphthyl-ethylenediamine was added and incubated for 5 min in the dark. The absorbance of the colored product was measured at 540 nm by ELISA.

A standard curve was performed in duplicate from a stock solution of NO_2_^−^ to establish a calibration curve for data analysis. Protein concentration was measured by the Bradford assay [60]. Results were expressed as µmol/l NO_2_^−^/mg protein.

### Assessment of NOS activity in oviductal cells

NOS activity was quantified by measuring the conversion of L-[^14^C]-Arginine to L-[^14^C]-citrulline, according to the technique described by Bredt and Snyder [61]. NO and L-citrulline are produced in equimolar amounts.

Briefly, confluent cell monolayers were incubated for 30 min at 38.5°C and 5% CO_2_ in the presence of: 1) M199 medium (control) or 2) MetAEA 1.4 nM. Control and treated cells were removed by trypsinization (Trypsin 25%+0.02% EDTA) and centrifuged at 1500 g for 5 min. Pellets were resuspended in a buffer containing 20 mM HEPES, 4.5 µM CaCl_2_ and 100 mM DTT and 25 mM valine, 10 µM [^14^C]-arginine (0.3 µCi) and 0.12 mM NADPH were added. Samples were incubated for 15 min in a 5% CO_2_ atmosphere at 37°C and immediately centrifuged at 28980 g for 10 min (4°C). Then the supernatants were applied to 1 ml DOWEX AG50W-X8 columns (Na^+^ form) equilibrated with HEPES medium containing 0.2 mM citrulline. Finally [^14^C]-citrulline was eluted in 2.5 ml of water. [^14^C]-L-citrulline radioactivity was measured by liquid scintillation counting. Protein concentration was measured by the Bradford assay [60]. Enzyme activity was expressed as pmoles [^14^C]-L-citrulline/mg protein/15 min.

### Immunocytochemistry

Spermatozoa were fixed (5 min, RT, 0.2% w/v paraformaldehyde), immobilized on slides and permeabilized with cold methanol as described in [23].

Non-specific binding sites were blocked (60 min, 10% v/v normal goat serum) and slices were treated with primary NOS antibodies (1∶50). Afterwards, samples were incubated with Alexa555-conjugated goat anti-rabbit IgG (1∶2000).

Specificity of the immunodetection was assessed by omitting the first antibody or by the replacement of specific primary antibody by serum from non-immunized rabbits at the same concentration (NOSs). Sperm cells were mounted and examined under a confocal laser imaging system (Nikon C1; Plan Apo 60/0.95, Japan).

### Statistical analysis

Data were analyzed by GLM procedures of one-way ANOVA (Di Rienzo J.A., Casanoves F., Balzarini M.G., Gonzalez L., Tablada M., Robledo C.W. InfoStat version 2010. Grupo InfoStat, FCA, Universidad Nacional de Córdoba, Argentina). Raw data were analyzed by Shapiro-Wilks and Levene tests to assess normality of data distribution and variance homogeneity, respectively. These procedures were applied in all variance analyses. Pairwise comparisons of means were made with Tukey or Fisher honestly significant differences. Results are expressed as mean±s.e.m of at least three independent determinations.

Fluorescence data are expressed as mean fluorescence (percentage of control, control adjusted to 100%). As these data did not follow a normal distribution a non parametric Kruskal Wallis analysis of variance was performed. Differences were considered significant when p<0.05 or less.

## References

[1] Suarez SS (2008). Regulation of sperm storage and movement in the mammalian oviduct.. Int J Dev Biol.

[2] Hunter RHF (2008). Sperm release from oviduct epithelial binding is controlled hormonally by peri-ovularoty Graafian follicles.. Mol Reprod Develop.

[3] Visconti PE, Westbrook VA, Chertihin O, Demarco I, Sleight S (2002). Novel signaling pathways involved in sperm acquisition of fertilizing capacity.. J Reprod Immunol.

[4] Herrero MB, de Lamirande E, Gagnon C (2003). Nitric oxide is a signaling molecule in spermatozoa.. Curr Pharm Des.

[5] Talevi R, Gualtieri R (2001). Sulfated glycoconjugates are powerful modulators of bovine sperm adhesion and release from the oviductal epithelium in vitro.. Biol Reprod.

[6] Gualtieri R, Boni R, Tosti E, Zagami M, Talevi R (2005). Intracellular calcium and protein tyrosine phosphorylation during the release of bovine sperm adhering to the fallopian tube epithelium in vitro.. Reproduction.

[7] Parrish JJ, Suako-Parrish J, Winer MA, First NL (1988). Capacitation of bovine sperm by heparin.. Biol Reprod.

[8] Parrish JJ, Susko-Parrish JL, Handrow RR, Ax RL, First NL (1989). Effect of sulphated glycoconjugates on capacitation and the acrosome reaction of bovine and hamster spermatozoa.. Garnet Res.

[9] Wang H, Dey SK (2005). Lipid signaling in embryo implantation.. Prostaglandins Other Lipid Mediat.

[10] Chun JY (2010). Lysophosphatidic acid (LPA) signaling in vertebrate reproduction.. Endocrinol Metab.

[11] Devane WA, Dysarz FA, Johnson MR, Melvin LS, Howlett AC (1988). Determination and characterization of a cannabinoid receptor in rat brain.. Mol Pharmacol.

[12] Munro S, Thomas KL, Abu-Shaar M (1993). Molecular characterization of a peripheral receptor for cannabinoids.. Nature.

[13] Ross RA (2003). Anandamide and vanilloid TRPV1 receptors.. Br J Pharmacol.

[14] Di Marzo V, Fontana A, Cadas H, Schinelli S, Cimino G (1994). Formation and inactivation of endogenous cannabinoid anandamide in central neurons.. Nature.

[15] Maccarrone M, Barboni B, Paradisi A, Bernabo N, Gasperi V (2005). Characterization of the endocannabinoid system in boar spermatozoa and implications for sperm capacitation and acrosome reaction.. J Cell Sci.

[16] Sun X, Dey SK (2008). Aspects of endocannabinoid signaling in periimplantation biology.. Mol Cell Endocrinol.

[17] Francavilla F, Battista N, Barbonetti A, Vassallo MR, Rapino C (2009). Characterization of the endocannabinoid system in human spermatozoa and involvement of transient receptor potential vanilloid 1 receptor in their fertilizing ability.. Endocrinology.

[18] Aquila S, Guido C, Laezza C, Santoro A, Pezzi V (2009). A new role of anandamide in human sperm: focus on metabolism.. J Cell Physiol.

[19] Schuel H, Burkman LJ, Lippes J, Crickard K, Mahony MC (2002). Evidence that anandamide-signaling regulates human sperm functions required for fertilization.. Mol Reprod Dev.

[20] El-Talatini MR, Taylor AH, Konje JC (2009). The relationship between plasma levels of the endocannabinoid, anandamide, sex steroids, and gonadotrophins during the menstrual cycle.. Fertil Steril.

[21] Amoako AA, Marczylo TH, Lam PM, Willets JM, Derry A (2010). Quantitative analysis of anandamide and related acylethanolamides in human seminal plasma by ultra performance liquid chromatography tandem mass spectrometry.. J Chromatogr B Analyt Technol Biomed Life Sci.

[22] Gervasi MG, Rapanelli M, Ribeiro ML, Farina M, Billi S (2009). The endocannabinoid system in bull sperm and bovine oviductal epithelium: role of anandamide in sperm-oviduct interaction.. Reproduction.

[23] Gervasi MG, Osycka-Salut C, Caballero J, Vazquez-Levin M, Pereyra E (2011). Anandamide capacitates bull spermatozoa through CB1 and TRPV1 activation.. PLoS One.

[24] Howlett AC (2005). Cannabinoid receptor signaling.. Handb Exp Pharmacol.

[25] Demuth DG, Molleman A (2006). Cannabinoid signalling.. Life Sci.

[26] Palmer RM, Moncada S (1989). A novel citrulline-forming enzyme implicated in the formation of nitric oxide by vascular endothelial cells.. Biochem Biophys Res Commun.

[27] Bartlett SR, Bennett PR, Campa JS, Dennes WJ, Slater DM (1999). Expression of nitric oxide synthase isoforms in pregnant human myometrium.. J Physiol.

[28] Törnblom SA, Maul H, Klimaviciute A, Garfield RE, Byström B (2005). mRNA expression and localization of bNOS, eNOS and iNOS in human cervix at preterm and term labour.. Reprod Biol Endocrinol.

[29] Lapointe J, Roy M, St-Pierre I, Kimmins S, Gauvreau D (2006). Hormonal and spatial regulation of nitric oxide synthases (NOS) (neuronal NOS, inducible NOS, and endothelial NOS) in the oviducts.. Endocrinology.

[30] Herrero MB, Pérez Martínez S, Viggiano JM, Polak JM, de Gimeno MF (1996). Localization by indirect immunofluorescence of nitric oxide synthase in mouse and human spermatozoa.. Reprod Fertil Dev.

[31] Herrero MB, Goin JC, Boquet M, Canteros MG, Franchi AM (1997). The nitric oxide synthase of mouse spermatozoa.. FEBS Lett.

[32] Meiser H, Schulz R (2003). Detection and localization of two constitutive NOS isoforms in bull spermatozoa.. Anat Histol Embryol.

[33] Perez Martinez S, Viggiano M, Franchi AM, Herrero MB, Ortiz ME (2000). Effect of nitric oxide synthase inhibitors on ovum transport and oviductal smooth muscle activity in the rat oviduct.. J Reprod Fertil.

[34] de Lamirande E, O'Flaherty C (2008). Sperm activation: role of reactive oxygen species and kinases.. Biochim Biophys Acta.

[35] Hou ML, Huang SY, Lai YK, Lee WC (2008). Geldanamycin augments nitric oxide production and promotes capacitation in boar spermatozoa.. Anim Reprod Sci.

[36] Roessner C, Paasch U, Glander HJ, Grunewald S (2010). Activity of nitric oxide synthase in mature and immature human spermatozoa.. Andrologia.

[37] Rodriguez PC, O'Flaherty CM, Beconi MT, Beorlegui NB (2005). Nitric oxide-induced capacitation of cryopreserved bull spermatozoa and assessment of participating regulatory pathways.. Anim Reprod Sci.

[38] Signorello MG, Giacobbe E, Passalacqua M, Leoncini G (2011). The anandamide effect on NO/cGMP pathway in human platelets.. J Cell Biochem.

[39] Herradón E, Martín MI, López-Miranda V (2007). Characterization of the vasorelaxant mechanisms of the endocannabinoid anandamide in rat aorta.. Br J Pharmacol.

[40] Vercelli CA, Aisemberg J, Billi S, Cervini M, Ribeiro ML (2009). Anandamide regulates lipopolysaccharide-induced nitric oxide synthesis and tissue damage in the murine uterus.. Reprod Biomed Online.

[41] Poblete IM, Orliac ML, Briones R, Adler-Graschinsky E, Huidobro-Toro JP (2005). Anandamide elicits an acute release of nitric oxide through endothelial TRPV1 receptor activation in the rat arterial mesenteric bed.. J Physiol.

[42] Cella M, Leguizamón GF, Sordelli MS, Cervini M, Guadagnoli T (2008). Dual effect of anandamide on rat placenta nitric oxide synthesis.. Placenta.

[43] Maccarrone M (2008). CB2 receptors in reproduction.. Br J Pharmacol.

[44] Revah I, Suarez SS, Flesch FM, Colenbrander B, Gadella BM (2000). Physiological state of bull sperm affects fucose- and mannose-binding properties.. Biol Reprod.

[45] Rodriguez PC, Beconi MT (2009). Peroxynitrite participates in mechanisms involved in capacitation of cryopreserved cattle.. Anim Reprod Sci.

[46] O'Flaherty C, Rodriguez P, Srivastava S (2004). L-arginine promotes capacitation and acrosome reaction in cryopreserved bovine spermatozoa.. Biochim Biophys Acta.

[47] Lefebvre R, Suarez SS (1996). Effect of capacitation on bull sperm binding to homologous oviductal epithelium.. Biol Reprod.

[48] Smith TT, Yanagimachi R (1991). Attachment and release of spermatozoa from the caudal isthmus of the hamster oviduct.. J Reprod Fertil.

[49] Fazeli A, Duncan AE, Watson PF, Holt WV (1999). Sperm-oviduct interaction: induction of capacitation and preferential binding of uncapacitated spermatozoa to oviductal epithelial cells in porcine species.. Biol Reprod.

[50] Bosch P, de Avila JM, Ellington JE, Wright RW (2001). Heparin and Ca^2+^- free medium can enhance release of bull sperm attached to oviductal epithelial cell monolayers.. Theriogenology.

[51] Rosselli M, Dubey RK, Rosselli MA, Macas E, Fink D (1996). Identification of nitric oxide synthase in human and bovine oviduct.. Mol Hum Reprod.

[52] Ghasemi M, Sadeghipour H, Dehpour AR (2007). Anandamide improves the impaired nitric oxide-mediated neurogenic relaxation of the corpus cavernosum in diabetic rats: involvement of cannabinoid CB1 and vanilloid VR1 receptors.. BJU Int.

[53] Fukui Y, Sonoyama T, Mochizuki H, Ono H (1990). Effects of heparin dosage and sperm capacitation time on in vitro fertilization and cleavage of bovine oocytes matured in vitro.. Theriogenology.

[54] Ward CR, Storey BT (1984). Determination of the time course of capacitation in mouse spermatozoa using a chlortetracycline fluorescence assay.. Dev Biol.

[55] Lee MA, Trucco GS, Bechtol KB, Wummer N, Kopf GS (1987). Capacitation and acrosome reactions in human spermatozoa monitored by a chlortetracycline fluorescence assay.. Fertil Steril.

[56] Galantino-Homer HL, Visconti PE, Kopf GS (1997). Regulation of protein tyrosine phosphorylation during bovine sperm capacitation by a cyclic adenosine 3′5′-monophosphate-dependent pathway.. Biol Reprod.

[57] Cross NL, Morales P, Overstreet JW, Hanson FW (1986). Two simple methods for detecting acrosome-reacted human sperm.. Gamete Res.

[58] Kojima H, Nakatsubo N, Kikuchi K, Kawahara S, Kirino Y (1998). Detection and imaging of nitric oxide with novel fluorescent indicators: diaminofluoresceins.. Anal Chem.

[59] Grisham MB, Johnson GG, Lancaster JR (1996). Quantitation of nitrate and nitrite in extracellular fluids.. Methods Enzymol.

[60] Bradford MM (1976). A rapid and sensitive method for the quantitation of microgram quantities of protein utilizing the principle of protein-dye binding.. Anal Biochem.

[61] Bredt DS, Snyder SH (1989). Nitric oxide mediates glutamate-linked enhancement of cGMP levels in the cerebellum.. Proc Natl Acad Sci U S A.

